# From High-Tech To High-Risk? Unveiling the Acute Ecotoxicological Effects of Rare Earth Elements on *Daphnia magna*

**DOI:** 10.1007/s00128-025-04044-7

**Published:** 2025-04-26

**Authors:** Ivo Pinto, Bruno Henriques, Thainara Viana, Rosa Freitas, Eduarda Pereira, Sara C. Antunes

**Affiliations:** 1 ICBAS, Instituto de Ciências Biomédicas de Abel Salazar, Universidade do Porto, Rua de Jorge Viterbo Ferreira, 228, 4050-313 Porto, Portugal; 2 CIIMAR/CIMAR LA, Interdisciplinary Centre of Marine and Environmental Research, University of Porto, Terminal de Cruzeiros do Porto de Leixões, 4450-208 Matosinhos, Portugal; 3 UMIB-ICBAS, Unidade Multidisciplinar de Investigação Biomédica - Instituto Ciências Abel Salazar da Universidade do Porto, Rua de Jorge Viterbo Ferreira no. 228, 4050-313 Porto, Portugal; 4 FCUP, Departamento de Biologia, Faculdade de Ciências, Universidade do Porto, Rua do Campo Alegre S/N, 4169-007 Porto, Portugal; 5 LAQV-REQUIMTE - Associated Laboratory for Green Chemistry, Department of Chemistry, University of Aveiro, 3810-193 Aveiro, Portugal; 6 Department of Biology, CESAM - Centro de Estudos do Ambiente e do Mar, University of Aveiro, 3810-193 Aveiro, Portugal

**Keywords:** Ecotoxicity, Lanthanoids, Transition metals, Cladocera, Acute toxicity

## Abstract

Technological advancement has brought significant environmental challenges, as its associated waste is difficult to manage and its long-term effects on ecosystems and biota remains uncertain. This study aimed to evaluate the acute toxicity of rare earth elements (REEs): lanthanum, cerium, praseodymium, neodymium, samarium, europium, gadolinium, terbium, dysprosium, holmium, erbium, thulium, ytterbium, lutetium, scandium, and yttrium on the standard aquatic species *Daphnia magna* through a toxicity screening approach. Based on EC_50_ values, the most toxic element was yttrium (EC_50_ = 7.2 mg L^−1^), followed by scandium, dysprosium, europium, lutetium, neodymium, holmium, gadolinium, ytterbium, thulium, terbium, samarium, cerium, and lanthanum (10 > EC_50_ < 100) identified as harmful, and praseodymium classified as non-toxic with an EC_50_ above 100 mg L^−1^ (130.81 mg L^−1^). This study demonstrates that all REEs, except praseodymium, induce acute effects in *D. magna* using ASTM as a culture medium. The results also highlight the need for standardized screening studies to obtain reliable data for both predictive and retrospective environmental risk features.

## Introduction

In recent decades, rapid technological advancements have made electronic and electrical devices essential to modern society, significantly increasing the demand of rare earth elements (REEs) (Henriques et al. 2021). This urge in consumption has also led to an exponential and unsustainable rise in electronic waste (e-waste) (Forti et al. 2020), which includes batteries, printed circuit boards, cathode ray tubes, computers, mobile phones, and televisions, among others (Frazzoli et al. 2022). Since 2014, global e-waste generation has grown by 9.2 Mt and is expected to reach 74.7 Mt by 2030, nearly doubling in just 16 years (Baldé et al. 2022). Outdated and improper disposal methods, such as open burning of printed circuit boards, contribute to severe environmental contamination by releasing toxic substances, including furans, dioxins, trace metals, and REEs (Pascale et al. 2018). The REEs include a group of 15 lanthanides: lanthanum (La), cerium (Ce), praseodymium (Pr), neodymium (Nd), promethium (Pm), samarium (Sm), europium (Eu), gadolinium (Gd), terbium (Tb), dysprosium (Dy), holmium (Ho), erbium (Er), thulium (Tm), ytterbium (Yb), and lutetium (Lu); and the transition metals: scandium (Sc) and yttrium (Y) (Egler et al. 2022). REEs occur naturally in the Earth’s crust and are present in marine environments, rivers, and lakes at concentrations ranging from ng L^−1^ to µg L^−1^ (Migaszewski and Gałuszka 2015). However, extensive industrial use and mining have led to elevated REE levels in various environmental matrices (Arciszewska et al. 2022; Hissler et al. 2014).

Several studies have demonstrated that REEs can have significant biological and ecological effects, including inhibited growth, developmental malformations, immobilization, and biomarkers response. These impacts have been observed in various aquaticspecies, such as *Aliivibrio fischeri*, *Raphidocelis subcapitata*, *Ulva lactuca*, *Fucus vesiculosus*, *Brachionus calyciflorus*, *Daphnia magna*, *Crassostrea gigas*, and *Danio rerio* (Andrade et al. 2023, 2024; Henriques et al. 2021; Moreira et al. 2020; Romero-Freire et al. 2019) as well as in terrestrial organisms (e.g., Egler et al. 2022). However, the concentrations of REEs required to induce these effects vary significantly among species (Agathokleous et al. 2018; Vignati et al. 2024; Wang et al. 2012). These variations have been reported by several authors and are attributed to differences in culture media and REEs speciation, as well as the physicochemical properties of the medium (e.g., pH) (Blinova et al. 2018; Lachaux et al. 2023; Vukov et al. 2016). Moreira et al. (2020) evaluated the effects of La and Y on the embryonic development of oyster *C. gigas* and found that La was the most toxic, with an EC_50_ ranging from 6.7 to 36.1 µg L^−1^, while Y exhibited intermediate toxicity with an EC_50_ between 147.0 and 221.9 µg L^−1^. Leite et al. (2024) assessed the impacts of different concentrations of Pr and Eu (0, 10, 20, 40, and 80 µg L^−1^) in the mussel species *Mytilus galloprovincialis*, showing that mussels’ response was dependent on the element. The authors noted that mussels’ defense mechanisms prevented lipid peroxidation when exposed to Pr, except at the highest concentration. However, protein carbonylation occurred at intermediate concentrations, indicating insufficient defense. In contrast, cellular damage was observed in mussels exposed to all Eu concentrations, reflecting limited defense capacity against this element.

Recognizing the impact of REEs on different environmental matrices, is urgent to conduct environmental risk assessments, either retrospective, where the problem has already been identified, or predictive, to anticipate potential consequences arising from the use and improper disposal of waste containing these elements. This is essential for developing more effective and targeted mitigation measures. Therefore, it is necessary to obtain ecotoxicological data to support the various tiers of environmental risk assessment. With the identification of environmental risk (Tier 0), the main objective of this study was to generate data to support Tier 1 (provides an initial overview of the risk, often using conservative assumptions and simplified models to determine if further investigation is needed) of the ecological risk assessment for REEs. In this context, an acute ecotoxicological screening of *Daphnia magna* after exposure to REEs was conducted. *D. magna* is a microcrustacean considered a standard organism in aquatic ecotoxicology (OECD 2012) due to high sensitivity to several compounds (e.g., metals, pesticides, PAHs) (Antunes et al. 2004, 2007, 2010; de Oliveira et al. 2016; Masteling et al. 2016).

## Methods and Materials

### Chemicals and Test Solutions

The lanthanoids and transition metals (Table 1) used to assess the acute toxicity were acquired in the format of chloride hexahydrate salt (XCl_3_·6H_2_O, where X is the chemical element, Sigma-Aldrich). Individual stock solutions of each element were prepared by dissolving the respective REE in ultrapure water (18 MΩ cm^−1^) (Table 1).

**Table 1:** Properties of each REE, stock solutions (mg L^−1^), and range of concentrations tested (mg L^−1^); 4.6 ≤ pH ≤ 5.0

Chemical element	CAS	Salt molecular weight (g mol^−1^)	Purity (%)	Stock solutions (mg L^−1^)	Concentration assay (mg L^−1^)
Lanthanum (La)	10025-84-0	371.37	98.0	1300	95–115
Cerium (Ce)	18618-55-8	372.58	99.9	1500	90–110
Praseodymium (Pr)	19423-77-9	247.27	99.9	6000	110–500
Neodymium (Nd)	13477-89-9	358.69	99.9	1200	50–90
Samarium (Sm)	10361-82-7	256.72	99.9	1000	80–100
Europium (Eu)	13759-92-7	366.41	99.9	1000	5–100
Gadolinium (Gd)	13450-84-5	371.70	99.9	1300	10–100
Terbium (Tb)	13798-24-8	373.38	99.9	1500	80–100
Dysprosium (Dy)	15059-52-6	376.95	99.9	600	18–44
Holmium (Ho)	4914-84-2	379.38	99.9	1000	25–80
Erbium (Er)	10025-75-9	381.71	99.9	1000	30–80
Thulium (Tm)	1331-74-4	383.38	99.9	1000	75–95
Ytterbium (Yb)	10035-01-5	387.49	99.9	1000	55–80
Lutetium (Lu)	7439-94-3	174.97	99.9	950	35–100
Scandium (Sc)	12060-08-1	137.91	99.9	500	7.5–40
Yttrium (Y)	7440-65-5	88.91	99.9	135	2–20

### *Daphnia magna* Culture Maintenance

*D. magna* cultures were continuously kept in controlled laboratory conditions for successive generations (more than 20 years). Monoclonal cultures were maintained in the synthetic water medium, “ASTM hard water” (ASTM 1980; Baird et al. 1989), a low-salt medium with a chemical composition per litre of: 192 mg of NaHCO_3_ (CAS: 144-55-8) and 120 mg of MgSO_4_·7 H_2_O (CAS: 10034-99-8); 8 mg of KCl (CAS: 7447-40-7); and 120 mg of CaSO_4_·2 H_2_O (CAS: 10101-41-4). It is a hard water medium (final hardness of 160–180 mg CaCO_3_ L^−1^), with a final pH of 7.0–7.5, supplemented with a combined vitamin solution, including 75 µg L^−1^ of thiamine, 1 µg L^−1^ of biotin, and 0.75 µg L^−1^ of cyanocobalamin. In culture, the daphniids were renewed every two days and fed with the microalga *Raphidocelis subcapitata* at a rate of 3 × 10^5^ cells mL^−1^day^−1^. The microalga was cultured in nonaxenic batch cultures with Woods Hole MBL medium under controlled continuous light (~ 6000 lx), temperature (20 ± 2 °C), and with aeration (Rodrigues et al. 2021). *D. magna* cultures were also supplemented with a standard organic additive, *Ascophyllum nodosum* extract (Antunes et al. 2007; Baird et al. 1988). The cultures were maintained in a culture chamber (Incubator TC 445 S, Lovibond^®^ Water Testing) under controlled conditions of 16 h^L^:8 h^D^ photoperiod and a temperature of 20 ± 2 °C.

### *Daphnia magna* Acute Assay

The standard guideline 202 of the OECD (2004) was used to perform the *D. magna* acute immobilization assay. A control group (with ASTM) and a range of concentrations (5 to 6) were prepared for each REE. For each concentration and control, four replicates were prepared in glass vessels (pre-washed with 25% HNO_3_ for 24 h) with 27 mL of each test solution, and 5 organisms (less than 24 h old, born between the 3rd and 5th broods) were added. The assay was performed under controlled conditions following a protocol similar to those used for culture maintenance (Sect. 2.2). The assay was conducted for 48 h under a static design, and at the end of the exposure period, the dead or immobilized organisms were counted for further determination of EC_50_ (48 h) values and corresponding confidence intervals at 95%. The immobilization of the daphniids refers to the absence of any movement for 15 s following a gentle agitation of the test vessel (OECD 2004). The validity criterion of the *D. magna* assay was an immobilization percentage lower than 10% in negative controls To assess potential losses or contamination, “chemical” blanks were always performed in parallel, consisting of ASTM medium fortified with REEs, in the absence of *D. magna*. The addition of each REE to the medium caused small changes in pH– the mean pH, measured after 48 h, was 7.4 ± 0.3 (the lowest values ​​were observed for the highest concentrations of REEs and the highest pH values for the lower concentrations of REEs).

The estimation of EC_50_ values and respective confidence intervals (CIs 95%) of *D. magna* for each tested compound were performed by modeling immobilization as binomial data (using the R package “drc”; (Ritz and Streibig 2005), with a special case of the log-logistic dose-response model, where the asymptotes of the curve are fixed to be 1 (all organisms are immobilized) and 0 (none are immobile), following the rationale of Ritz (2010).

### Analytical Concentrations Determination

For each element, the quantification of analytical concentrations was performed at the beginning (0 h) and at the end (48 h) of the assay. A volume of 1 mL of the assay medium was collected from the glass vessels and 25 µL of HNO_3_ was immediately added to ensure a pH < 2. Quantification of total REEs was performed by Inductively Coupled Plasma Optical Emission Spectroscopy (ICP-OES) using a Horiba Jobin Yvon, Activa M. Calibration curves were obtained with multi-element standards ranging from 10 µg L^−1^ to 100 mg L^−1^ (coefficient determination ≥ 0.999). The limit of quantification was assumed as the lowest calibration standard and the maximum acceptable variation among replicates was 5%. Blank measurements were always carried out between samples.

## Results and Discussion

The concentrations of REEs measured in the medium samples after spiking were in agreement with those expected. Table 2 shows, as an example, the real exposure concentrations for La, which are representative of the light rare earth elements and had the smallest deviations from the nominal concentrations (1%); Gd, which represents heavy rare earth elements; and for Nd, which showed the largest deviations with respect to the nominal concentrations (29%). The deviations from the initial concentration, assessed by blank assays, were always less than 18% during the 48 h exposure, which indicates that potential element loss (e.g., byprecipitation and subsequent deposition or adsorption to vessel walls) was not relevant and that REEs were relatively stable in solution. One of the primary challenges in studying REEs ecotoxicity in standard ecotoxicological media is the formation of insoluble precipitates. These precipitates reduce the bioavailability of REEs to organisms, potentially leading to an underestimation of their toxicity (Lachaux et al. 2023). The precipitation of REEs is influenced by their speciation and is promoted by higher pH values and increased REEs concentrations (Blinova et al. 2018). Vignati et al. (2024), in their work exposed several REEs to *D. magna* and observed that by decreasing the pH of the culture medium to 6.5 after 48 h only 20% of the element was lost, unlike when they used a pH of 7.8. REEs carbonates are easy to precipitate due to the low solubility of REEs-carbonate complexes, particularly at neutral to basic pH (González et al. 2015; Lachaux et al. 2023). REEs nitrates hydrolyze easily in solution, especially at higher concentrations, which results in complexation or precipitation (Blinova et al. 2018). A simulation of the speciation of the REEs in ASTM was performed with Visual MINTEQ 3.1 under the experimental conditions used in this work and the results show that 100% of the elements will be in the dissolved fraction, mostly in the form of complexes with carbonate (45 to 69% as LnCO_3_^+^), sulfate (13 to 30% as LnSO_4_^+^) and in its free ionic form (6 to 18% as Ln^3+^– Table 3).

**Table 2:** Nominal and real exposure concentrations of La (light rare earth element), Gd (heavy rare earth element), and Nd (element with the largest deviations), (*n* = 4), at 0 h and 48 h. pH = 7.4 ± 0.3

Element	Nominal concentration (mg L^−1^)	0 h		48 h	
		Real concentration (mg L^−1^)	Deviation from nominal concentration (%)	Final concentration (mg L^−1^)	Deviation from nominal concentration (%)
La	95	95.0 ± 12	0	87.0 ± 5.0	8
	105	106 ± 15	1	92.0 ± 1.0	13
	110	111 ± 21	1	91.0 ± 3.0	18
	115	115 ± 14	0	104 ± 3.0	10
Gd	10	11.0 ± 1.0	6	10.0 ± 0.0	9
	20	22.0 ± 1.0	12	23.0 ± 0.0	−5
	40	44.0 ± 1.0	9	43.0 ± 0.0	2
	80	90.0 ± 2.0	12	88.0 ± 1.0	2
	100	110 ± 4.0	10	109 ± 6.0	1
Nd	50	61.0 ± 1.0	21	59.0 ± 1.4	3
	60	75.0 ± 0.0	25	73.0 ± 1.4	3
	70	90.0 ± 0.0	29	84.0 ± 4.2	7
	80	100 ± 0.0	25	100 ± 0.0	0
	90	115 ± 7.1	28	110 ± 3.5	4

**Table 3:** Distribution of La, Gd, and Nd species in the exposure medium for the highest concentration tested - simulations performed in visual MINTEQ 3.1 software, using the chemical composition of the culture medium as input

Element	% of total concentration	Species name
La^3+^	17.82	La^3+^
	0.258	LaOH^2+^
	0.083	LaCl^2+^
	1.277	La(SO_4_)_2_^−^
	30.59	LaSO_4_^+^
	3.376	LaHCO_3_^2+^
	1.808	La(CO_3_)_2_^−^
	44.78	LaCO_3_^+^
Gd^3+^	6.336	Gd^3+^
	1.188	GdHCO_3_^2+^
	0.889	GdOH^2+^
	0.014	GdCl^2+^
	0.468	Gd(SO_4_)_2_^−^
	13.112	GdSO_4_^+^
	8.645	Gd(CO_3_)_2_^−^
	69.35	GdCO_3_^+^
Nd^3+^	8.414	Nd^3+^
	0.524	NdOH^2+^
	0.456	Nd(SO_4_)_2_^−^
	16.64	NdSO_4_^+^
	1.247	NdHCO_3_^2+^
	5.091	Nd(CO_3_)_2_^−^
	67.62	NdCO_3_^+^

Regarding the literature focused on REEs toxicity, significant discrepancies were observed in toxicity values, with several authors indicating that toxicity can be attributed to external (abiotic) factors (Barry and Meehan 2000; Herrmann et al. 2016; Lachaux et al. 2022; Malhotra et al. 2020; Revel et al. 2023). Some studies reported that variation in the composition of the culture medium influences the toxicity levels of La, Nd, Gd, and Yb in *D. magna* (Barry and Meehan 2000; Blinova et al. 2020; Lachaux et al. 2022; Vukov et al. 2016). The use of REE chlorides in the ASTM medium in this study demonstrated a certain degree of chemical stability, with variations of less than 30% from the initial nominal concentrations (Table 2). Several factors may contribute to this increased stability. One possible explanation is that the ASTM medium has a more stable chemical composition ensuring the physiological conditions of *Daphnia* (Olkova 2022). Additionally, the lower hardness of ASTM, compared to the OECD medium, may enhance the bioavailability of heavy metals, including REEs, by minimizing their precipitation (Okamoto et al. 2015; Revel et al. 2025). Furthermore, the ASTM medium has a more acidic pH (7.0–7.5) compared to the OECD medium (pH = 7.8) or to the M4 medium (7.5–8.5), which results in lower formation of insoluble rare earth element hydroxides (REE(OH)_3_). This occurs because these compounds tend to precipitate as the pH approaches to 8.0–8.5 values. Therefore, at pH levels close to 7, most of the REEs remain dissolved, increasing their bioavailability and potential toxicity to *D. magna*. Specifically, higher pH levels promote the formation of insoluble hydroxides, reducing their availability in the medium. Malhotra et al. (2020) and Revel et al. (2023) also showed that different pH values change the bioavailability and distribution of REEs influencing the effects on organisms. Given these complexities, comparing and evaluating the effects of various REEs is challenging and must be approached with caution to prevent overestimating or underestimating their impact.

**Fig. 1:**
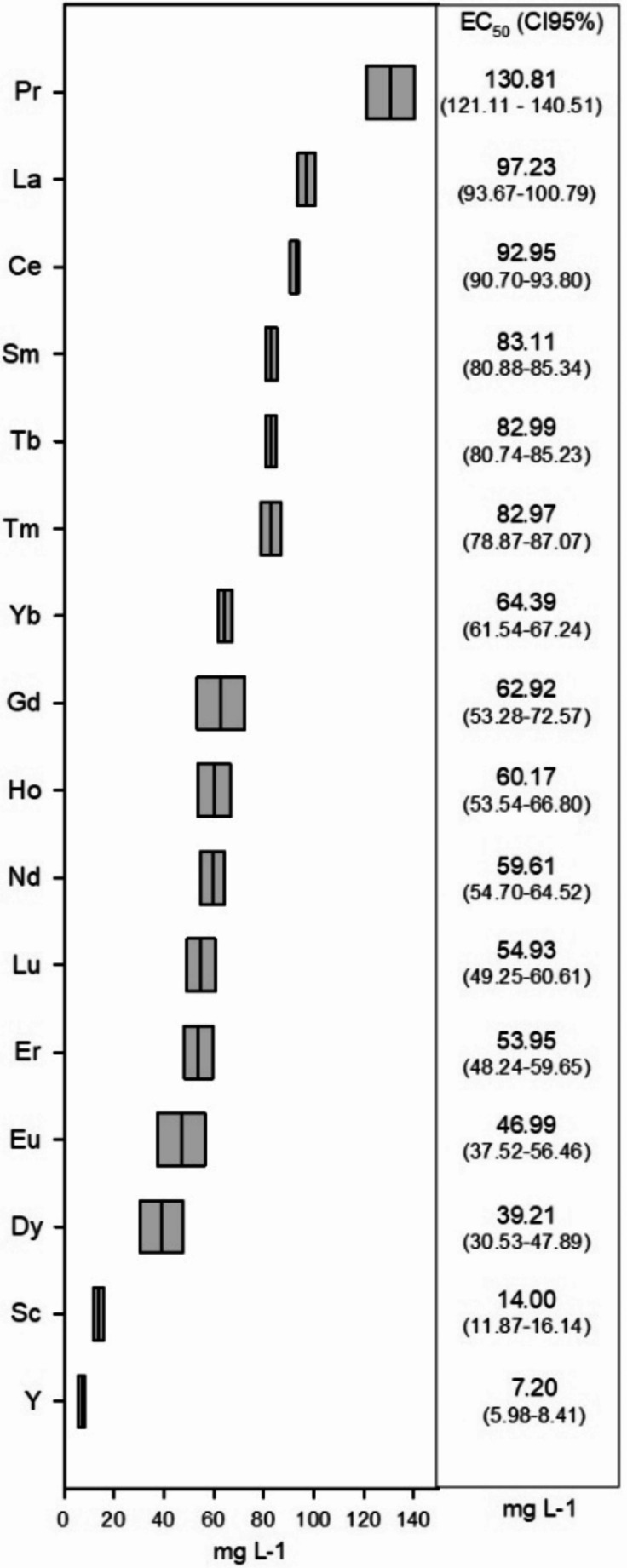
EC_50_ values (mg L^−1^) and correspondent confidence intervals at 95% for *D. magna* after acute exposure (48 h) to each REE tested

Based on the EC_50_ values determined for the REEs tested (Fig. 1), the elements Pr, La, and Ce present the highest EC_50_ values (130.81, 97.23, and 92.95 mg L^−1^, respectively), which demonstrate that these elements have lower toxicity when compared to the remaining REEs analyzed. On the other hand, the elements Y, Sc, and Dy presented low EC_50_ values (7.20, 14.00, and 39.21 mg L^−1^, respectively), showing that they are the most toxic elements to *D. magna* regarding acute exposure (48 h). Following the ecotoxicological classification of Annex VI of Directive 67/548/EECl, the REEs tested revealed different toxicological effects: only Y showed to be toxic (EC_50_ ≤ 10 and > 1 mg L^−1^) while Pr showed to be non-toxic (EC_50_ ≥ 100 mg L^−1^). The remaining REEs were considered harmful (EC_50_ > 10 and < 100 mg L^−1^) regarding the *D. magna* acute toxicity values recorded (Fig. 1).

Regarding the effects on *Daphnia* sp., Blinova et al. (2018) exposed *D. magna* to Praseodymium nitrate (Pr(NO_3_)_3_·6H_2_O) for 48 h and obtained an EC_50_ = 23.8 mg L^−1^, a value much lower and not corroborating the non-toxic classification observed in the present work (EC_50_ [PrCl_3_·6H_2_O] = 130.81 mg L^−1^; Fig. 1), however, we are unable to understand the reason for these discrepancies. Regarding La, one of the most studied compounds (Barry and Meehan 2000; Egler et al. 2023; Korkmaz et al. 2021; Revel et al. 2023), in the present study it was the second least toxic REEs with a EC_50_ of 97.23 mg L^−1^ (Fig. 1). Barry and Meehan (2000) exposed *Daphnia carinata* to La using three different media and obtained EC_50_ values around 0.050 mg L^−1^ using soft tap water or diluted sea water, and an EC_50_ of 1.18 mg L^−1^using ASTM hard water. Once again, and based on the discrepancy in results, it is possible to observe that the composition of the media influences the EC_50_ values. On the other hand, using an ASTM medium at similar conditions as our assay, the discrepancy in values is most likely due to the use of a different *Daphnia* species, showing that different species may exhibit varying responses and sensitivities to the same element. Egler et al. (2023) exposed *Daphnia similis* in MS medium to La_2_O_3_ using the metal solution and obtained an EC_50_ of 17.61 mg L^−1^. As mentioned by the authors, the MS medium contained ethylenediaminetetraacetic acid (EDTA) and cyanocobalamin as chelators, which can complex with the REEs and render them non-bioavailable to the tested organisms. Korkmaz et al. (2021) performed an assay using *D. magna* in natural water and observed a 60% mortality at a concentration of 50 mg L^−1^ of La, however, the study does not provide information about the culture medium, nor in what format the element was supplied, so it is not possible to understand what could justify the differences in the EC_50_ values. Another study by Revel et al. (2023) involving La and *D. magna*, in which they replaced NaCl for NaHCO_3_ to reduce/avoid precipitation, but without success, showed that only 8% of the organisms were immobile at 30 mg L^−1^, indicating that the EC_50_ value must be higher than this concentration. However, the La concentrations measured after the exposure were between 2 and 3 mg L^−1^, except for the highest nominal concentration (30 mg L^−1^) where the measured concentration was 0.46 mg L^−1^ highlighting the relevance of studying all external factors to perceive the real REEs toxicity.

Gadolinium, Nd, and Yb showed middle toxicity values according to our results (Fig. 1; EC_50_ between 59 and 65 mg L^−1^); however, they exhibit higher values compared to the literature. Lachaux et al. (2022) used Gd chloride, Nd nitrate, and Yb chloride to assess the acute toxicity of *D. magna* and observed similar toxicity values of these compounds (EC_50_ ranged between 8.3 and 8.8 mg L^−1^ for the three compounds). Despite the toxicity values, these results corroborate our results where similar toxicity values were also recorded between these REEs (Fig. 1). Egler et al. (2023) when exposed *D. similis* to Nd_2_O_3_ using the MS medium obtained an EC_50_ of 25.43 mg L^−1^, a lower value than that obtained in the present work (Fig. 1), however, we have already reinforced the limitations of this medium (Egler et al. 2023). Furthermore, it is noteworthy that the authors reported that Nd is more toxic than Sm, and Sm is more toxic than La, ranking the elements in the same order as observed in the present study (Fig. 1). However, regarding the elements that showed higher toxicity to *D. magna* in this study − Y, Sc, and Dy (EC_50_ < 40 mg L^−1^) − no research studies with acute exposure to *Daphnia* sp. were found in the literature. Cardon et al. (2019) recorded a 100% mortality rate in *D. magna* after exposure to 1.187 mg Y L^−1^, but only after 7 days.

The findings of this study highlight the importance of accounting for environmental factors, such as medium composition and pH, when assessing REEs toxicity. These factors directly affect bioavailability and, in turn, influence environmental risk assessment. With the ongoing use of REEs and their improper disposal in the environment, it is anticipated that their concentration will increase, particularly in storage areas for this equipment. This rise may eventually reach levels observed in this study, which have been shown to impact key species in freshwater aquatic ecosystems. The discrepancy in EC_50_ values obtained in literature reinforces the need for a careful approach to risk analysis, avoiding under or overestimations that could compromise the effectiveness of mitigation measures. The data presented here serves as a fundamental basis for Tier 1 of ecological risk assessment, enabling an initial estimation of the potential impacts of REEs in a controlled laboratory context. Additionally, it provides valuable guidance for further studies, supporting subsequent tiers of environmental risk assessment and contributing to more effective environmental management.

## Conclusion

An acute toxicity screening in ASTM hard water of the total REEs concentrations was performed, showing that Y was the most toxic element (EC_50_ = 7.20 mg L^−1^) and Pr the least toxic (EC_50_ = 130.81 mg L^−1^) for *Daphnia magna*. Additionally, this work provides an acute toxicity screening for a wide range of lanthanides using a standard methodology, enabling better comparison and interpretation of data between studies and facilitating the inference of REEs effects. The increase in concentrations of REEs in water bodies is a current concern, and the present work is essential to understand the effects on model species such as *D. magna*, providing an initial overview of the risk, often using conservative assumptions and simplified models to determine if further investigation is needed. Investigations on long-time exposure, different species, bioaccumulation, and biochemical effects of REEs can also be useful for better understanding their ecotoxicity.
